# Tricuspid Valve-in-Valve Procedure with An Edwards S3 Valve in a 15-kg Child in Latin America

**DOI:** 10.21470/1678-9741-2021-0557

**Published:** 2023

**Authors:** Albert Franz Guerrero Becerra, Jaime Ramon Cabrales Arevalo, Julian Senosiain, Alberto Enrique Garcia Torres, Jaime Camacho, Nestor Fernando Sandoval Reyes

**Affiliations:** 1 Department of Cardiac Surgery, Fundación Cardioinfantil – Instituto de Cardiología, Bogotá, Cundinamarca, Colombia.; 2 Department of Interventional Cardiologist, Fundación Cardioinfantil – Instituto de Cardiología, Bogotá, Cundinamarca, Colombia.; 3 Fundación Cardioinfantil – Instituto de Cardiología, Bogotá, Cundinamarca, Colombia.

**Keywords:** Tricuspid Valve Disease, Congenital Heart Disease, Transcatheter Valve Implantation, Sternotomy, Child

## Abstract

A 5-year-old child, weighing 15 kg, with three previous sternotomies, presented with right heart failure due to severe stenosis and regurgitation of the bioprosthetic tricuspid valve. A percutaneous tricuspid valve-in-valve procedure with an Edwards S3 valve was ofered for compassionate use, performed with no complications and with a significant clinical condition improvement.

## CASE PRESENTATION

In 2018, a two-year-old male patient was brought in from a rural area in Colombia due to cyanotic congenital heart disease. He had multiple episodes of respiratory infections in addition to cyanosis on crying or physical exertion. On admission to Fundación Cardioinfantil, an echocardiogram was performed revealing a double outlet right ventricle (DORV), side-by-side vessels, a subaortic ventricular septal defect (VSD), narrowed subaortic cone without gradient, and a large persistent ductus arteriosus with severe pulmonary hypertension. In May 2018, the patient was referred for surgical correction of DORV along with unrelated VSD closure with tunneled Dacron patch, disinsertion of the tricuspid papillary septal muscle and reimplantation in the Dacron patch, patent ductus arteriosus closure and tricuspid valve repair.

In July 2018, the patient was readmitted due to stage C, Stevenson B decompensated heart failure. A new echocardiogram showed a grade III tricuspid regurgitation. He was again referred for surgery and a tricuspid valvuloplasty was performed using an autologous pericardial patch. One week following surgical correction, severe tricuspid regurgitation was observed. As a result, the tricuspid valve was replaced using a 25-mm Perimount biological prosthesis. Outpatient follow-up was done throughout the 2018-2019 period. The patient had a successful recovery, and no cardiovascular symptoms were observed.

He was readmitted in October 2020 due to a fall from own height along with granuloma formation at the surgical site, mild dyspnea, tachypnea and occasional cough. Echocardiogram revealed severe stenosis of the biological tricuspid prosthesis with a mean gradient of 12-16 mmHg. The patient was referred for balloon valvuloplasty. At the end of the procedure, a grade 2-3 biological valve insufficiency was observed.

## TECHNICAL DESCRIPTION

In this situation, a new surgery was ruled out at the heart team meeting, and a percutaneous tricuspid valve-in-valve procedure was ofered for compassionate use. Informed consent was obtained from the parents. The procedure followed the guidelines of the local ethics committee.

**Fig. 1:**
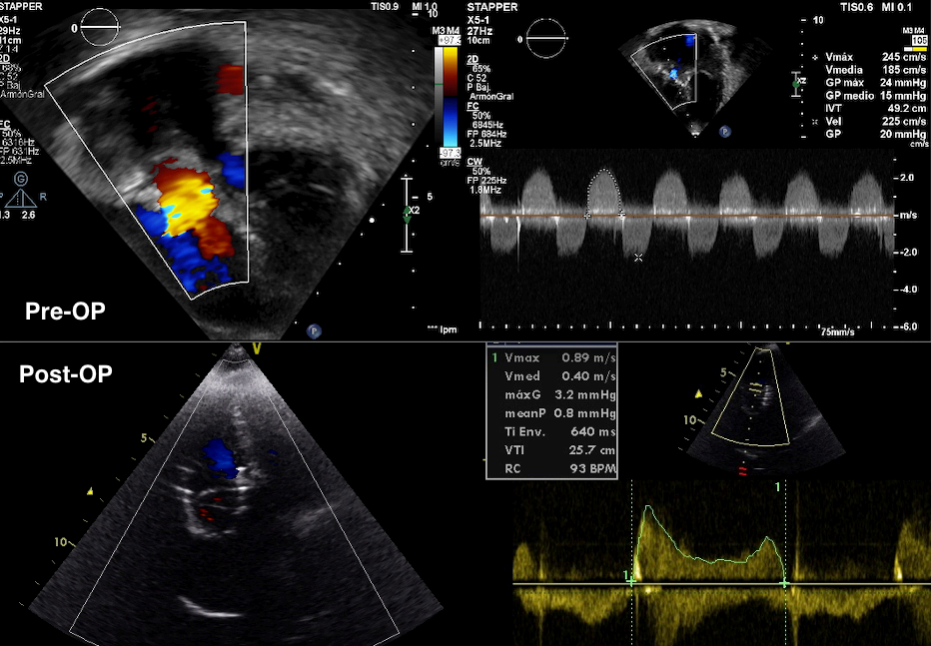
Pre-OP: tricuspid valve gradient before implantation. Post-OP: tricuspid valve gradient 1 year after surgery.

**Fig. 2:**
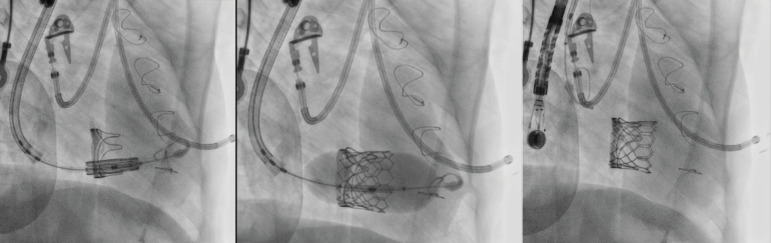
Valve implantation exposed in a sequence.

In October 2020, the procedure was performed in the hybrid operating room under general anesthesia and using echocardiography (Figure 1) to assess valve function as well as guiding implantation. The right jugular vein was punctured after echographic evaluation, with an 8-French sheath. The right femoral artery was punctured with a 5-French sheath for invasive blood pressure monitoring. Intravenous antibiotics and heparin were administered. Through the 8-French introducer, a 6-French JR 3.5 catheter was advanced, once positioned, the 14-French eSheath was placed, then the CONFIA guidewire was introduced and left in position in the right ventricle. We chose the 26-mm Edwards S3 valve (Edwards Lifesciences, Inc, Irvine, CA, USA) mounted on the balloon. Finally, the new valve was successfully positioned and implanted (Figure 2). No paravalvular leak or valve regurgitation was observed on transesophageal echocardiography. We decided to place a 26-mm Edwards S3 valve since the previous one was a 25-mm Perimount and the effective orifice for this valve was adequate.

The patient remained in the intensive care unit for the first 24 hours, being discharged 4 days after admission, with improvement in the right heart failure symptoms, and on warfarin therapy for three months and antiplatelet therapy after completing the three months of anticoagulation. Two years later, the valve continues to perform well, without stenosis (mean gradient 2 mmHg) or regurgitation, and the patient is asymptomatic (NYHA I).

## COMMENT

Tricuspid valve dysfunction is a relatively uncommon occurrence, with higher prevalence in individuals with congenital heart abnormalities, often involving complex patients^[1]^.

Severe tricuspid valve dysfunction and especially severe regurgitation are associated with increased mortality regardless of other factors. Surgical tricuspid valve replacement is the main indication for the treatment of severe tricuspid valve dysfunction (regurgitation, stenosis or mixed disease)^[2,3]^. In the tricuspid position, bioprosthetic valves are generally preferred over mechanical valves, given the failure rates and anticoagulation-associated complications. However, these bioprosthetic valves undergo a gradual^[2,3]^ degeneration requiring successive replacements. Management of these patients is complicated by the presence of previous sternotomies and high surgical morbidity and mortality, which makes a percutaneous approach an appealing option^[2,3]^. The implantation of percutaneous valves in the tricuspid position is still an of-label indication, but it can be an option to avoid reinterventions in tricuspid bioprosthesis. Here, we describe a case of successful percutaneous tricuspid valve-in-valve procedure, with a 26-mm Edwards Sapien S3© valve in a 15 kg boy. To the best of our knowledge, this is the smallest patient in which a tricuspid valve-in-valve procedure with an Edwards Sapien valve has been performed in Latin America.

Sapien and Melody© valves (Medtronic Inc, Minneapolis, MN, USA) have been used in tricuspid valve-in-valve procedures^[4]^, but Melody valve is significantly longer than Sapien, even though the valve can be cut to reducing its length^[5]^ it was an important issue in a 15 kg patient.

Given the small size of our patient, with a small part of the sheath inside him, short distance and sharp curve between the sheath and the target, and an adequate diameter of the jugular vein, anticipating the possibility of difficulties in this step, we preferred to use a 14F eSheath, allowing to mount the Sapien valve on the balloon outside the patient, and reducing manipulation inside him.

McElhinney et al. reported a multicenter registry with 152 cases of tricuspid valve-in-valve procedures with Melody and Sapien valves, with a medium-term follow-up. Successful implantation was performed in 150 patients, with a 30-day mortality of 3.3% (all NYHA III–IV at baseline). Estimated reintervention-free survival was 85±3% at 1 year, with NYHA IV, baseline renal failure, and in-hospital acutely ill as statistically significant risk factors. All centers in the registry performed 1 to 3 procedures, showing that tricuspid valve-in-valve procedure appears to be technically straightforward and reproducible across a large number of centers despite the small volume, and confrming that the risk-benefit profile of tricuspid valve-in-valve procedures^[3]^ appear to be technically straightforward and reproducible across a large number of centers despite the small volume, and confrming that the risk-benefit profile of tricuspid valve-in-valve procedures is generally favorable. Percutaneous tricuspid valve-in-valve is a valuable option for treating complex patients with severe symptomatic tricuspid valve dysfunction, even in very small patients, and ofers the possibility of delaying and shortening surgical procedures throughout the lives of these patients. Unfortunately, there are no multicentric studies, nor medium-and long-term studies evaluating the durability and degree of degeneration of these prostheses at this age.
